# Rural Community Perspectives on Hurricane Helene in Western North Carolina

**DOI:** 10.13023/jah.0801.05

**Published:** 2026-04-01

**Authors:** Jennifer A. Horney, Lilly Moreau, SEARCH WNC

**Affiliations:** University of Delaware; University of Delaware; Sustaining Essential and Rural Community Healthcare

**Keywords:** Appalachia, Community Resilience, Disaster, Hurricane Helene, Rural, Western North Carolina

## Abstract

**Introduction:**

After making landfall on the Florida Gulf Coast in September 2024, Hurricane Helene impacted Western North Carolina (WNC) with historic flooding, landslides, and hurricane-force winds. More than 100 deaths in the region were attributed to the storm’s direct impacts.

**Purpose:**

The purpose of this study was to assess community perspectives of long-term recovery in Mitchell and Yancey Counties, two rural WNC counties severely impacted by Hurricane Helene.

**Methods:**

A 14-question interview guide was developed based on prior disaster research and the community engagement work of Sustaining Essential and Rural Community Healthcare (SEARCH). Questions covered post-Helene experiences related to access to food, shelter, transportation, and health care as well as the successes and challenges to the coordination of multiple groups around hurricane response and recovery. Key informants in the two counties were invited to participate in an in-person interview. Recordings were inductively coded to identify themes.

**Results:**

Five themes were identified through analysis of 16 interview transcripts. These included (1) housing issues exacerbated by the storm, (2) challenges with communication during and after the storm, (3) challenges with transportation, (4) mental health impacts, and (5) food insecurity.

**Implications:**

As inland flooding associated with wetter tropical cyclones becomes more severe, the populations at risk of health and other impacts of this flooding will grow. Even in areas well-prepared and well-resourced for emergency response, the failure of essential infrastructure such as communications and transportation will limit effectiveness of response and recovery. Rural areas are at particular risk due to limited alternatives such as public transportation, affordable short-term housing, and limited access to broadband and Wi-Fi.

## INTRODUCTION

Rural communities face unique challenges after a disaster that are often the result of overlapping vulnerabilities related to limited housing and transportation, employment or income loss, and fewer emergency response and recovery resources.^1^,^2^ For example, when Hurricane Michael made landfall in a rural area along Florida’s (FL) panhandle, most residents reported home damage, loss of income, and difficulty accessing healthcare and necessary medications that persisted more than five months after the hurricane.^3^ In rural areas of Texas impacted by Hurricane Harvey, housing availability and affordability were both more impacted when compared to urban areas in part because of limits on redevelopment and transition of housing stock to short-term vacation rentals.^4^ In rural areas of Eastern North Carolina (ENC) impacted by Hurricane Matthew, flood damage to rural homes and schools was associated with problems obtaining asthma medications for children, missing doses, or running out of medications.^5^

Hurricane Helene made landfall in FL as a Category 4 storm, the strongest to impact the Big Bend region of that state since at least 1900.^6^ Simultaneously, Western North Carolina (WNC) was experiencing significant rainfall from another storm system. As Hurricane Helene tracked across the Southeastern U.S., it remained a significant wind and rain event with impacts to WNC starting September 27, 2024. Although by the time the storm arrived in Central Appalachia it had been downgraded to a tropical storm, we refer to the incident as Hurricane Helene, while recognizing that it is an inaccurate reflection of the storm when it arrived in the region. The area experienced historic flooding, landslides, and hurricane-force winds^6^ impacting public communication, transportation, and other infrastructure closing many local businesses, and destroying thousands of homes. Individual and public assistance were made available by the State of North Carolina and the Federal Emergency Management Agency (FEMA) to address: the loss of housing, the needed repairs to private roads and bridges, disaster-related, unemployment, debris removal, small business loans, restored access to safe water and sewer systems, and crisis counseling and behavioral health interventions.^7^

In May 2025, the North Carolina Office of Management and Budget estimated the direct and indirect impacts of Helene in WNC to be approximately $60 billion.^7^ More than 100 deaths were attributed to the hurricane’s direct impacts, which included a near total disruption of critical infrastructure, including communications, in some cases for months.

### Purpose

The purpose of this project was to collect qualitative information from key informants in two rural counties of WNC to describe Hurricane Helene’s impacts and the ongoing recovery as well as to inform the development of a quantitative household survey^8^ to collect data on long-term recovery.

## METHODS

Sustaining Essential and Rural Community Healthcare (SEARCH) is a nonprofit organization focused on advocating for access to sustained quality healthcare for the residents of Mitchell and Yancey Counties in North Carolina (NC). The two counties have a total population of about 34,000 residents spread over about 535 square miles. The University of Delaware’s Department of Epidemiology and SEARCH jointly developed a 14-question interview guide based on published hazards and disaster research, as well as prior community engagement and research activities of SEARCH that occurred prior to and continue in the aftermath of Helene. Questions included post-Helene experiences related to access to food, shelter, and transportation as well as the coordination of multiple groups around hurricane response and recovery. Given SEARCH’s mission, several questions focused on short-term access to physical and mental health services after Hurricane Helene as well as the longer-term implications of the disaster on the availability of these services for local residents.

City and County managers, local health department staff, healthcare facility staff, law enforcement personnel, nonprofit organization leaders, elected officials, and others active in the disaster recovery efforts in Mitchell and Yancey Counties, were identified and contacted by SEARCH to arrange a mutually agreeable time for the interview. The SEARCH volunteers’ familiarity with the local community and prior engagement with the key informants allowed for the establishment of trust and rapport and facilitated participation. Interviews were conducted in person and recorded with permission. In addition, detailed notes were taken by the interviewer. Notes and recordings were transcribed by two authors who inductively identified themes and met in person to resolve any discrepancies through discussion. The interview guide and all other documents were reviewed and approved by the University of Delaware’s Institutional Review Board (2291737-1).

## RESULTS

Sixteen interviews were completed with key informants across the two counties by SEARCH volunteers. An inductive analysis of the key informant interview transcripts identified five themes, including housing issues exacerbated by the storm, challenges with communication during and after the storm, challenges with transportation, mental health impacts, and food security.

### Housing Shortages and Safety

As one respondent stated, Hurricane Helene “made the housing shortage worse, particularly in Yancey County.” Although estimates vary, approximately 70,000 homes were damaged or destroyed across the region leaving many residents needing both temporary and longer-term housing, especially in terms of rental housing. In some disasters, short-term housing needs can be met with so-called “FEMA trailers” although their placement in flood-prone areas and their long-term use have been controversial after other disasters.^9^ As one interviewee stated,

“[E]ven if they are able to get a camper through whatever resources…there’s no place to put them. A lot of campgrounds were lost. Or they were renting before so they don’t have land that they can put a camper on.”

The lack of affordable housing makes other aspects of the recovery more difficult as well. For example, one key informant mentioned that “professionals who are being recruited to join the local healthcare workforce can’t find housing, so they choose to go elsewhere for work.” The potential health impacts of living in inadequately repaired homes is also a concern, with key informants pointing to “increases in allergy and asthma symptoms” from exposure to contaminants as well as concerns about “unknown long-term health impacts from mold.”

### Communication Barriers

The widespread and long-lasting outages of electricity, mobile phone networks, and landlines following Hurricane Helene were well publicized in the media. According to the Federal Communications Commission (FCC), several days after the storm more than 60% of cell sites were out of service in the impacted areas of NC; by October 13, 2024, that number was reduced to around 6%.^10^ However, the loss of communications had major impacts on emergency response with one interviewee stating that “the emergency plan did not really work in this situation...because the communication systems did not work.” The lack of communication also had impacts on other aspects of response and recovery. One respondent stated that “the area mostly had enough heavy equipment, but they couldn’t communicate to where it was needed,” which left many residents stranded. Another pointed out that “because there was no ability to communicate, it was easy for false information to spread.”

### Transportation Barriers

Damage to the transportation infrastructure in this area of rural WNC reverberated into many aspects of recovery. Housing shortages were intensified when “private roads were in need of repairs that [residents] were unable to afford, and private bridges were damaged or destroyed leaving residents unable to live in their homes.” Thousands of cars were flooded with many individuals “unable to purchase replacement vehicles” and “very limited public transportation.” Transportation barriers will also have long-term impacts on recovery, particularly among vulnerable groups like children and the elderly. For example, one respondent mentioned that “summer food distribution programs for children may not be possible due to road and bridges closures and the inability to get to people’s homes.” Another pointed out the disproportionate impacts of transportation disruptions on the elderly, who could not be reached by a “support system of people that could check on them or make sure they had the basic items they needed every day.”

### Mental Health Impacts

Post-traumatic stress disorder (PTSD) is one of the most well-studied post-disaster psychological disorders,^11^ and fear and emotional distress associated with experiences of severe weather are common, as are subsequent physical and psychological responses.^12^ In the case of Hurricane Helene, several key informants mentioned that people are “terrified of the rain and the wind.” Others pointed out that these fears have led children to “miss a lot of school, with some even having to repeat a grade.” One respondent also mentioned that this “loss [of time in school] adds to the losses during [the] COVID [pandemic]” and pointed out that even with community support, “there were going to be gaps.”

### Food Security

The availability of food was mentioned frequently in the interviews. Respondents were concerned about the short- and long-term impacts of the destruction of farms on the local food supply. As one respondent stated, “almost all the farms were covered in sand and farmers are reconsidering farming because it will take at least three to five years to restore.” Another pointed out that some local farms were “reducing the amount of land they were farming by up to 75%” due to the damages. Even residents who had plots in community gardens or home gardens that are relied on by “many to feed their families and neighbors alike,” were unable to repair the damage to plants after the storm. While prepared foods became available through the disaster response and volunteer efforts, “access to fresh and healthy foods, specifically produce, was a concern” for many residents. Many restaurants closed and according to one respondent, “Mitchell County lost two grocery stores; now there is only Walmart.”

## IMPLICATIONS

Long after the direct impacts of Hurricane Helene on rural WNC, much work remains as part of continued long-term recovery and resilience building. After-action reports and other assessments conducted by state and local authorities identified communication disruptions, which included specialized communication systems used by emergency responders necessary for the implementation of response as well as regular telephone and internet services, as a critical failure. As has been clearly demonstrated in prior research (e.g., after Hurricane Michael impacted rural FL), the restoration of cell phone and Internet services were cited as the most important factors related to returning to regular activities. For example, without the restoration of Internet services, key informants pointed out that rural WNC residents faced high barriers in completing online applications for disaster assistance.

It is also important to note that in the case of Hurricane Helene, the impact of the loss of communication included emergency response coordination, enabling the spread of disinformation and slowing access to recovery resources, both of which made it more difficult to address the needs of those impacted by disasters. In this case, refuting rumors took time and attention away from real issues due to the limited ability to staff emergency response offices around the clock and a shortage of cross-trained emergency management staff. Disinformation negatively affected residents’ trust in the response and recovery and politicized these processes.

While the generalizability of these findings is limited as only two of the 25 NC counties where a major disaster declaration was issued were included, because of their relatively small populations and limited infrastructure, the impacts of disasters and emergencies on rural communities can be proportionally greater.^13^ Other factors that may increase the impacts include lower rates of health, flood, and homeowners’ insurance, higher rates of poverty and unemployment, geographic isolation, and limited planning and administrative capacity. The restoration of services needed as part of emergency response, as well as the building of resilience during recovery, can also be hindered in rural areas for these same reasons. In rural WNC, the loss of housing, communication, and transportation as well as the short- and long-term impacts on physical and mental health and food security, remain a challenge for residents and local jurisdictions as the next hurricane season begins.

**SUMMARY BOX**

**What is already known about this topic?**

Rural areas are uniquely vulnerable to the impacts of disasters. Challenges to the continuity of communication and coordination are key disruptions that negatively impact a community’s ability to respond to a disaster.

**What is added by this report?**

Much of what is known about the impacts of Helene in Western North Carolina has been focused on the most urbanized area around Asheville. Inland and flash flooding from stronger and wetter tropical storms is a growing threat to residents of Appalachia.

**What are the implications for future research?**

While communication disruptions during disasters have been highlighted as a limitation of effective response for several decades, these findings highlight the newer issues of the role disinformation plays in impeding response, reducing trust, and politicizing disasters.
